# The Protective Role of *Bacteroides fragilis* in a Murine Model of Colitis-Associated Colorectal Cancer

**DOI:** 10.1128/mSphere.00587-18

**Published:** 2018-11-14

**Authors:** Yun Kyung Lee, Parpi Mehrabian, Silva Boyajian, Wei-Li Wu, Jane Selicha, Steven Vonderfecht, Sarkis K. Mazmanian

**Affiliations:** a Division of Biology & Biological Engineering, California Institute of Technology, Pasadena, California, USA; b Soonchunhyang Institute of Medi-Bio Science, Soonchunhyang University, Cheonan, Republic of Korea; c Center for Comparative Medicine, Beckman Research Institute, City of Hope, Duarte, California, USA; d Department of Physiology, College of Medicine, National Cheng Kung University, Tainan, Taiwan; The Jackson Laboratory for Genomic Medicine

**Keywords:** *Bacteroides fragilis*, Toll-like receptor 2, colitis-associated colorectal cancer, inflammation, polysaccharide A

## Abstract

Many patients with chronic inflammation of the gut, such as that observed in inflammatory bowel disease (IBD), develop colorectal cancer (CRC). Recent studies have reported that the development of IBD and CRC partly results from an imbalanced composition of intestinal microbiota and that intestinal inflammation in these diseases can be modulated by the microbiota. The human commensal Bacteroides fragilis is best exemplified playing a protective role against the development of experimental colitis in several animal disease models. In this study, we found that gut inflammation caused by dextran sulfate sodium (DSS) treatment was inhibited by B. fragilis colonization in mice. Further, we reveal a protective role of B. fragilis treatment against colon tumorigenesis using an azoxymethane (AOM)/DSS-induced model of colitis-associated colon cancer in mice and demonstrate that the decreased tumorigenesis by B. fragilis administration is accompanied by inhibited expression of C-C chemokine receptor 5 (CCR5) in the gut. We show direct evidence that the inhibition of tumor formation provided by B. fragilis in colitis-associated CRC animals was dependent on the production of polysaccharide A (PSA) from B. fragilis and that Toll-like receptor 2 (TLR2) signaling was responsible for the protective function of B. fragilis.

**IMPORTANCE** The incidence of colorectal cancer (CRC) is rapidly growing worldwide, and there is therefore a greater emphasis on studies of the treatment or prevention of CRC pathogenesis. Recent studies suggested that consideration of the microbiota is unavoidable to understand inflammation and tumorigenesis in the gastrointestinal tract. We demonstrate, using a mouse model of colitis-associated CRC, that human commensal B. fragilis protects against colon tumorigenesis. The protective role against tumor formation provided by B. fragilis is associated with inhibition of expression of the chemokine receptor CCR5 in the colon. The molecular mechanism for protection against CRC provided by B. fragilis is dependent on polysaccharide A production and is mediated by TLR2 signaling. Our results suggest that the commensal microorganism B. fragilis can be used to prevent inflammation-associated CRC development and may provide an effective therapeutic strategy for CRC.

## Abstract (precis)

The incidence of colorectal cancer (CRC) is rapidly growing worldwide, and there is therefore a greater emphasis on studies of the treatment or prevention of CRC pathogenesis. Recent studies suggested that consideration of the microbiota is unavoidable to understand inflammation and tumorigenesis in the gastrointestinal tract. We demonstrate, using a mouse model of colitis-associated CRC, that human commensal B. fragilis protects against colon tumorigenesis. The protective role against tumor formation provided by B. fragilis is associated with inhibition of expression of the chemokine receptor CCR5 in the colon. The molecular mechanism for protection against CRC provided by B. fragilis is dependent on polysaccharide A production and is mediated by TLR2 signaling. Our results suggest that the commensal microorganism B. fragilis can be used to prevent inflammation-associated CRC development and may provide an effective therapeutic strategy for CRC.

## INTRODUCTION

The recent rapid increase in colorectal cancer (CRC) incidence is a serious health problem worldwide, as it has one of the highest rates of cancer-related mortality (1). Although various efforts have been made, the development of CRC remains to be completely understood owing to its complexity. A major triggering factor for the development of CRC is chronic inflammation in the gut, such as that due to inflammatory bowel disease (IBD) (2–4). Although approximately half of patients with IBD develop CRC (5), the mechanisms underlying how chronic IBD leads to the generation of CRC are not fully understood. Among environmental factors, the microbiota is a major contributor to induction of chronic inflammation in the gut, and an imbalance of microbiota composition is strongly associated with the incidence and progression of CRC (6, 7). In addition, it was shown that treatment with certain bacteria relieved inflammatory reactions in cancerous cell lines and that oral administration of beneficial bacteria protected against colon pathogenesis in several animal models of colitis and CRC (8–11). The results of those studies suggest that the modulation of microbiota might be a good strategy to prevent or cure CRC, and uncovering how symbionts suppress inflammatory response warrants further investigation.

The microbiota in the gastrointestinal tract modulates host metabolism and shapes the immune system to support human health (12, 13). In addition, the intestinal microbiota has been implicated in promotion or regulation of several diseases, including intestinal and extraintestinal pathogenesis (14–16). To understand how gut bacteria play a critical role in human health and disease conditions, studies using animal model are necessary to reveal the defined function of microbiota *in vivo*. Through such studies, the protective mechanism of certain bacteria against intestinal inflammation has been identified and its importance was recognized (8, 17). It has been shown that Bacteroides fragilis, a prominent human commensal, prevents intestinal inflammatory diseases in animal with colitis. The immunomodulatory molecule polysaccharide A (PSA) produced by B. fragilis induces an anti-inflammatory immune response in intestinal tissue (18, 19). In CD45RB^hi^ T-cell transfer- or 2,4,6-trinitrobenzene sulfonic acid (TNBS)-induced murine models, B. fragilis was shown to protect against the development of colitis by inducing the production of the anti-inflammatory cytokine interleukin-10 (IL-10) (18, 19). PSA of B. fragilis confers its beneficial function via Toll-like receptor 2 (TLR2) signaling (20, 21) and limits pathological inflammation by inducing IL-10 production from regulatory T cells (19). The results of those studies indicate that the anti-inflammatory effect of B. fragilis is important for protection from colitis.

Although the mechanisms of B. fragilis in protecting against intestinal inflammatory conditions have been revealed, no study has examined the role of B. fragilis in the development of colitis-associated CRC. As the microbiota is a critical factor for both IBD and CRC, and as chronic IBD is one cause of CRC development, the purpose of our study was to determine whether B. fragilis also modulates the pathogenesis of CRC, which is important for developing new treatments or cure for CRC. In this study, we demonstrated that B. fragilis treatment prevented the development of acute dextran sulfate sodium (DSS)-induced colitis by partly inhibiting IL-1β and C-C chemokine receptor 5 (CCR5) expression. More importantly, we found that PSA of B. fragilis inhibited the development of colitis-associated CRC in an azoxymethane (AOM)/DSS-induced animal model and that its beneficial effect against colon tumorigenesis was mainly determined by TLR2 signaling. Our findings suggest that PSA from B. fragilis can be a new candidate for CRC protection and provide additional insight into the function of microbial factors in ameliorating chronic inflammation and tumorigenesis in the gastrointestinal tract.

## RESULTS

### Colonization with B. fragilis protects against inflammation in DSS-induced colitis.

**FIG 1:**
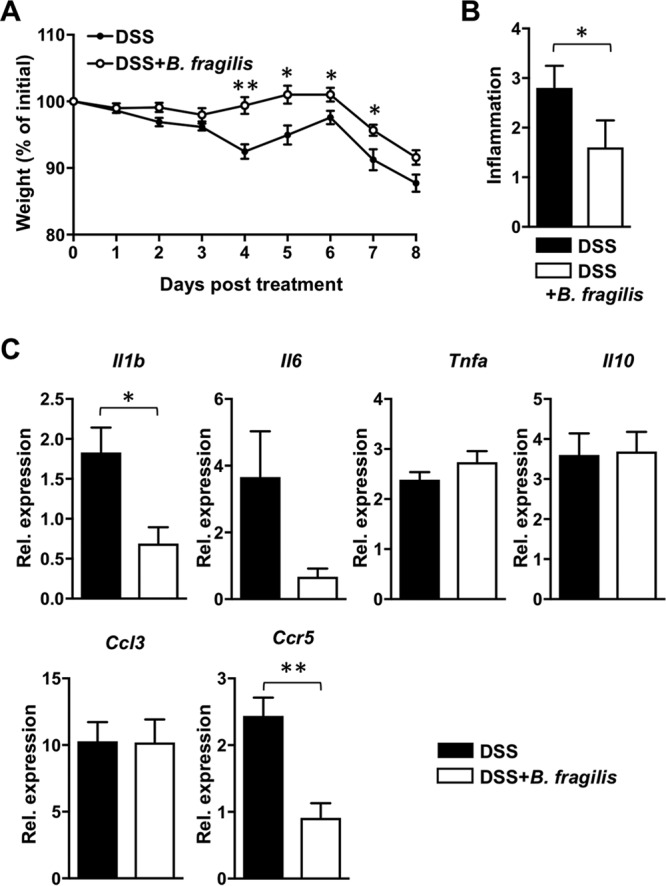
B. fragilis colonization protects mice against dextran sulfate sodium (DSS)-induced colitis. C57BL/6J (B6) mice were treated orally with either PBS or B. fragilis three times a week starting a week prior to DSS treatment and continuing until the end of the experiment (5 mice per group). DSS (2.5%) was administered via the drinking water for 6 days followed by administration of regular drinking water. Mice were sacrificed on day 8. (A) Percent weight change during DSS-induced colitis. (B) Inflammation score of colon sections of the indicated groups. (C) Expression levels of IL-1β, IL-6, TNF-α, IL-10, CCL3, and CCR5 from colon homogenates of control or B. fragilis-colonized mice on day 8 of DSS treatment. Relative values were normalized to that of β-actin. Data are representative of results from at least two experiments. Statistical significance was calculated using unpaired Student's *t* tests and nonparametric Mann-Whitney tests. *, *P < *0.05; **, *P < *0.005 (in all panels).

Previous studies have shown that the human commensal B. fragilis prevented the development of colitis in a T-cell transfer or TNBS model (18, 19); however, the role of B. fragilis in acute DSS-induced colitis has not been yet determined. Mice were colonized with B. fragilis by oral administration every other day starting 1 week before 2.5% DSS treatment. We observed that the weight loss of B. fragilis-colonized mice was significantly less than that of the control mice from day 4 to day 7 post-DSS treatment; however, the levels of weight loss on day 8 were comparable to control (Fig. 1A). In addition, mice with B. fragilis resisted weight loss until post-DSS treatment day 6 and started weight loss on day 7 under the DSS-treated condition. Histopathology staining revealed that mice with B. fragilis showed less-extensive pathological consequences on day 8 (see Fig. S1A in the supplemental material). Among the histopathology criteria, we found that the inflammation score of B. fragilis-treated mice was significantly lower than that of control DSS-treated mice (Fig. 1B), while there were no significant differences in the scores of ulceration, crypt loss, edema, and extent of injury on day 8 between the two groups (Fig. S1B). In a murine T-cell transfer or TNBS model of colitis, expression of IL-1β, IL-6, tumor necrosis factor alpha (TNF-α), and IL-10, which are involved in inflammation, was regulated by B. fragilis (18, 19, 22). Consistently, the expression of the proinflammatory cytokine IL-1β was downregulated by B. fragilis colonization in our animal model of DSS-induced colitis. Recent studies showed that CCR5 was responsible for recruitment of inflammatory cells in DSS-based models (23, 24). We observed decreased expression of CCR5 in B. fragilis-treated mice compared with the controls and comparable levels of expression of CCL3, a CCR5 ligand (Fig. 1C). Differential expression of IL-10, IL-6, and TNF-α was not observed in the B. fragilis-treated group with DSS administration, a finding which is different from the reported role of B. fragilis in preventing inflammation in T-cell transfer or a TNBS-induced colitis model. These results indicate that B. fragilis colonization protects against the development of colitis in a DSS-induced acute colitis mouse model and has different protective mechanisms under different colitis-induced conditions.

10.1128/mSphere.00587-18.1 FIG S1 B. fragilis colonization suppresses inflammation in dextran sulfate sodium (DSS)-induced colitis. C57BL/6J (B6) mice were treated orally with either PBS or B. fragilis three times a week starting a week prior to DSS treatment and continuing until the end of the experiment (5 mice per group). DSS (2.5%) was administered via the drinking water for 6 days followed by administration of regular drinking water. Mice were sacrificed on day 8. (A) Scores of ulceration, crypt loss, edema, and extent of injury from colon sections of the indicated groups. (B) Hematoxylin-and-eosin-stained representative section of colon from the indicated experimental group on day 8. Data are representative of results from at least two experiments. Statistical significance was calculated using nonparametric Mann-Whitney tests. Download FIG S1, PDF file, 1.0 MB. Copyright © 2018 Lee et al. 2018 Lee et al. This content is distributed under the terms of the Creative Commons Attribution 4.0 International license.

### Colonization with B. fragilis protects against development of colitis-associated CRC.

**FIG 2:**
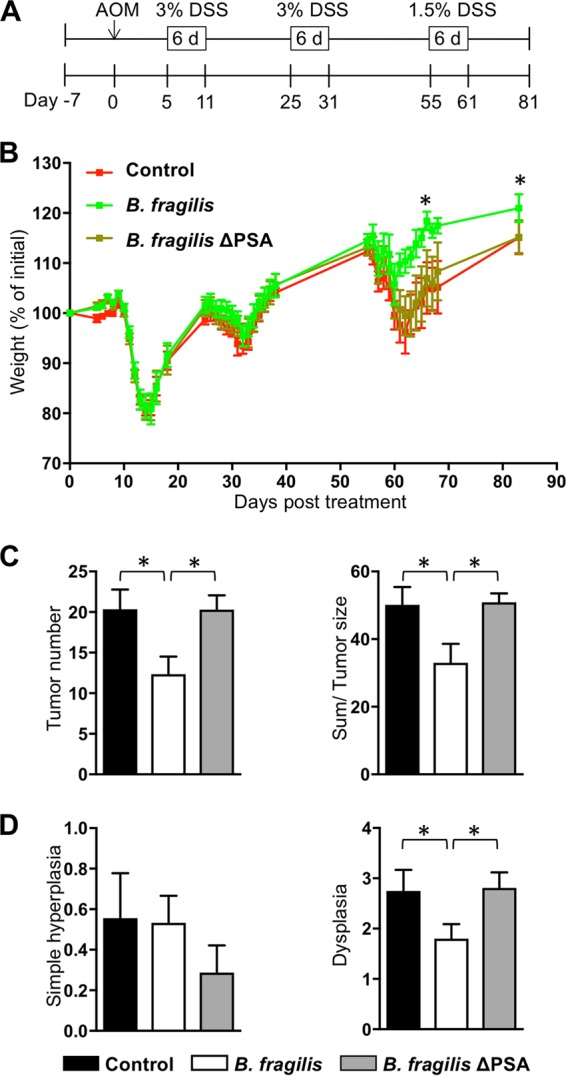
Colonization with B. fragilis protects mice against development of colon cancer. (A) Schematic overview of the azoxymethane (AOM)/dextran sulfate sodium (DSS)-induced colorectal cancer model. C57BL/6J (B6) mice were treated orally with PBS (control), B. fragilis, or B. fragilis ΔPSA three times a week starting a week prior to AOM injection until the end of the experiment (8 mice per group). After an initial AOM intraperitoneal injection (10 mg/kg), 3% DSS was administered via the drinking water for 6 days followed by administration of regular drinking water. The mice underwent a second DSS treatment cycle with 3% DSS-treated water on day 25 for 6 days and a third cycle with 1.5% DSS-treated water on day 55 for 4 to 6 days. The mice were sacrificed on day 81 post-AOM injection. (B) Percent weight change in the indicated groups during DSS treatment. (C) Number of tumors in the distal colon (left) and the sum of tumor size (right) in the indicated groups on day 81 of AOM-DSS treatment. (D) Scores of hyperplasia (left) and dysplasia (right) from longitudinal sections of distal colon of the indicated groups on day 81 of AOM-DSS treatment. Data are representative of results from at least two experiments. Statistical significance was calculated using unpaired Student's *t* tests and nonparametric Mann-Whitney tests. *, *P < *0.05 (in all panels).

Considering the high risk of CRC in patients with colitis (5), we hypothesized that mice treated with B. fragilis are less susceptible to colitis-associated CRC. AOM/DSS-induced colon cancer, representing a well-established colitis-associated CRC model, was used to examine the role of B. fragilis during the development of CRC. B. fragilis colonization started 1 week prior to AOM injection and continued until the end of experiment on day 81. Five days after AOM injection, the first phase of DSS treatment was started for 6 days and two additional DSS treatments were applied to the mice as described previously (25, 26). As weight loss is one of the indicators of disease progression, the body weights of mice were monitored during the three DSS-treatment periods (Fig. 2A). We found that the percentage of weight loss was significantly lower in the B. fragilis-treated group during the third DSS treatment phase and at the endpoint of the experiment than that seen with the controls (Fig. 2B). Moreover, the number and size of tumors in the colon of mice in the B. fragilis-treated group were significantly lower and smaller, respectively, than those in control mice (Fig. 2C). These findings indicate that colonization with B. fragilis protects against colon tumorigenesis induced with carcinogen/DSS.

Previous reports showed that PSA from B. fragilis is a critical factor in the prevention of several diseases such as chronic IBD and autoimmune diseases (18, 27, 28). Accordingly, the weight loss of mice treated with B. fragilis ΔPSA was comparable to that of controls during AOM-DSS treatment (Fig. 2B). The number of tumors and the tumor size in mice treated with B. fragilis ΔPSA were not reduced compared to those in mice treated with B. fragilis on day 81 of AOM-DSS treatment (Fig. 2C). These results suggest that PSA from B. fragilis is a critical factor in suppressing development of colitis-associated colon cancer. Our findings from histological analysis indicate that there were no differences in the results with respect to simple hyperplasia among the three groups. However, the dysplasia score for colon tissue in B. fragilis-treated mice was significantly lower than that in the control or B. fragilis ΔPSA-treated mice (Fig. 2D), a finding which indicates that PSA from B. fragilis suppresses colon cancer pathogenesis. Taken together, these findings indicate that B. fragilis protects against the development of colitis-associated CRC in a PSA-dependent manner.

### The protective role of B. fragilis colonization against colitis-associated CRC is associated with CCR5 expression.

**FIG 3:**
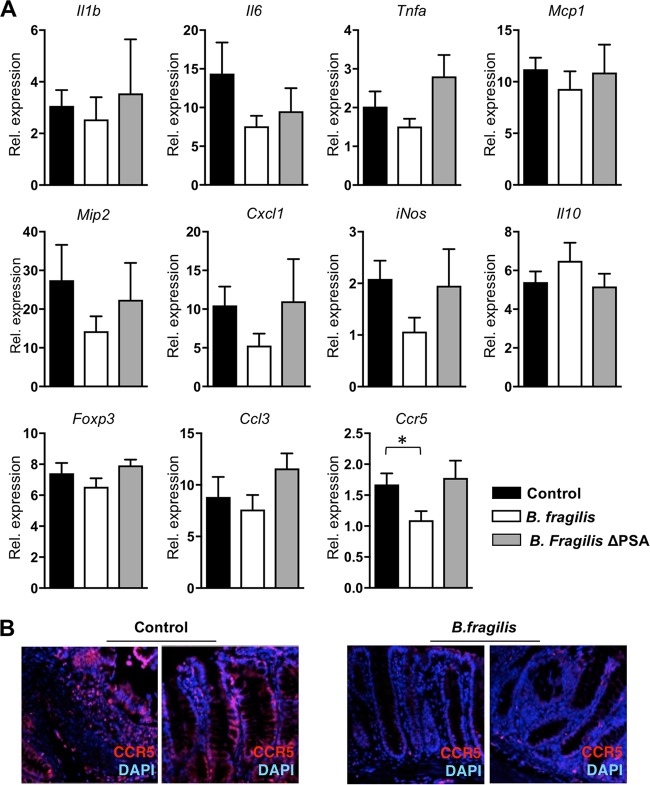
B. fragilis colonization regulates expression of proinflammatory cytokines and gene signatures associated with inflammation and tumor pathogenesis during colon cancer development in mice. (A) Expression levels of the indicated genes from colon homogenates of control, B. fragilis*-*colonized, or B. fragilis ΔPSA-colonized mice on day 81 of AOM-DSS treatment. Relative values were normalized to that of β-actin. (B) Immunofluorescent staining of CCR5 in colonic tissues of untreated control and B. fragilis*-*colonized mice on day 81 of AOM-DSS treatment. Data are representative of results from at least two experiments. Statistical significance was calculated using unpaired Student's *t* tests. *, *P < *0.05 (in all panels).

In addition to the tumorigenesis phenotype, we wished to determine the molecular mechanism underlying protection provided by B. fragilis against colitis-associated colon cancer. The genes of the inflammatory response- or colon cancer-related markers in colon homogenates from control, B. fragilis*-*treated, and B. fragilis ΔPSA-treated mice were analyzed on day 81 of AOM-DSS treatment. The levels of expression of proinflammatory cytokine genes *Il1b*, *Il6*, and *Tnfa* were measured. Key chemokines that are often present during inflammation, such as monocyte chemoattractant protein 1 (MCP-1), macrophage inflammatory protein 2 (MIP-2), and chemokine ligand 1 (CXCL1) (also known as KC), were also observed. The expression level of inducible nitric oxide synthase (iNOS), often observed in colon carcinogenesis, was also checked. We found that the expression levels of all these genes were not significantly different among the three groups, although all showed suppressed trends in the B. fragilis-treated group compared to the control or B. fragilis ΔPSA-treated groups (Fig. 3A). Further, as production of IL-10 from Foxp3-positive cells induced by B. fragilis is important for the protective role of B. fragilis in several disease animal models, expression of *Il10* and *Foxp3* was examined in control, B. fragilis*-*treated, and B. fragilis ΔPSA-treated groups. We observed that neither gene (*IL-10* or *Foxp3*) was induced by B. fragilis during colitis-associated CRC (Fig. 3A). However, decreased expression of CCR5, known to contribute to recruitment of inflammatory cells, was observed in colon tissue of mice treated with B. fragilis (Fig. 3), suggesting that reduced expression of the inflammatory and infiltration-inducing factor CCR5 is associated with the protective role of B. fragilis against the development of colitis-associated colon cancer.

### TLR2 is required for the B. fragilis-mediated protection against colon cancer.

**FIG 4:**
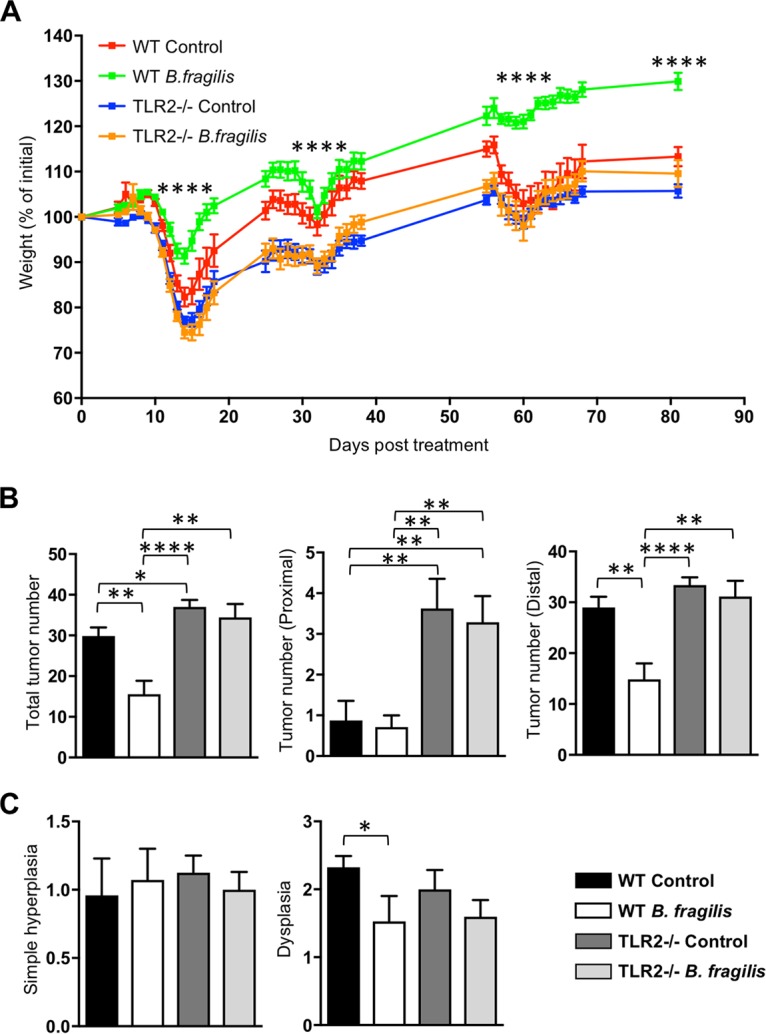
B. fragilis utilizes TLR2 signaling for its protective role against the development of colitis-associated cancer in mice. C57BL/6J (B6) or *Tlr2^−^*^/^*^−^* mice were treated orally with either PBS or B. fragilis three times a week starting a week prior to AOM injection until the end of the experiment (8 mice per group). After an initial AOM intraperitoneal injection (10 mg/kg), 2.5% DSS was administered via the drinking water for 6 days followed by administration of regular drinking water. The mice underwent a second DSS treatment cycle with 2.5% DSS-treated water on day 25 for 6 days and a third cycle with 1.5% DSS-treated water on day 55 for 4 to 6 days. The mice were sacrificed on day 81 post-AOM injection. (A) Percent weight change in the indicated groups during DSS treatment. WT, wild type. (B) Number of tumors in the total (left), proximal (middle), or distal (right) colon of the indicated groups on day 81 of AOM-DSS treatment. (C) Scores of hyperplasia (left) and dysplasia (right) from longitudinal sections of colon of the indicated groups on day 81 of AOM-DSS treatment. Data are representative of results from at least two experiments. Statistical significance was calculated using unpaired Student's *t* tests and nonparametric Mann-Whitney tests. *, *P < *0.05; **, *P < *0.005; ****, P* < *0.0001 (in all panels).

It was reported previously that TLR2 signaling is important for the anti-inflammatory response of B. fragilis in intestinal inflammation (20) and that TLR2 plays a protective role against the development of colitis-associated CRC (25). Thus, we next determined whether the TLR2 signaling pathway is responsible for B. fragilis protection against development of colitis-associated CRC. Wild-type C57BL/6J (B6) or *Tlr2^−^*^/^*^−^* mice were colonized with B. fragilis and treated with AOM-DSS during day 81, similarly to our previous experimental condition shown in Fig. 2A. DSS (2.5%) was applied at the first and second DSS periods for this experiment, as only half of the *Tlr2^−^*^/^*^−^* mice survived to the end of the experiment when 3% DSS was used. Under these low-DSS conditions, development of colitis-associated colon cancer in B6 mice was, as found in the previous, more aggressive protocol, significantly inhibited by B. fragilis colonization. The difference in the percentage of weight loss under this condition between mice from the B6 control group and B. fragilis-colonized was greater than that seen under the previous condition (3% DSS). Comparisons of tumor number and dysplasia score yielded results similar to our previous findings (Fig. 4).

In addition, compared to B6 control mice, TLR2-deficient mice that received AOM-DSS treatment significantly lost weight during the three phases of DSS treatment and at the endpoint of the experiment (Fig. 4A). Further, the number of tumors in TLR2-deficient mice was higher than the number in B6 mice, with an especially higher number of tumors found at the proximal colon of TLR2-deficient mice (Fig. 4B). This is consistent with a previous result showing that TLR2-deficient mice were susceptible to colitis-associated CRC (25). We also examined the expression of cytokines and gene signatures associated with inflammation and tumor pathogenesis of colon tissues and found that *Il10* expression was not significantly induced by B. fragilis in either wild-type or *Tlr2^−^*^/^*^−^* mice. Previous studies have suggested that the severity of the TLR2-deficient condition is mediated by STAT3 signaling (25), and we observed no significant differences in the expression levels of *Il6* and *Il23p19* in colon homogenates among the groups. Further, the downregulated CCR5 expression seen upon B. fragilis colonization was not observed in the TLR2-deficient mice, suggesting that the inhibition of CCR5 expression in B. fragilis-treated mice was not mediated by TLR2 (Fig. S2).

10.1128/mSphere.00587-18.2 FIG S2 Expression of proinflammatory cytokines and gene signatures associated with inflammation and tumor pathogenesis during colon cancer development in mice was regulated by B. fragilis treatment. C57BL/6J (B6) or *Tlr2^−^*^/^*^−^* mice were treated orally with either PBS or B. fragilis three times a week starting a week prior to AOM injection and continuing until the end of the experiment (8 mice per group). After an initial AOM intraperitoneal injection (10 mg/kg), 2.5% DSS was administered via drinking water for 6 days followed by administration of regular drinking water. The mice underwent a second DSS treatment cycle with 2.5% DSS-treated water on day 25 for 6 days and a third cycle with 1.5% DSS-treated water on day 55 for 4 to 6 days. The mice were sacrificed on day 81 post-AOM injection. Expression levels of the indicated genes in colon homogenates of the indicated groups on day 81 of AOM-DSS treatment are shown. Relative values were normalized to that of β-actin. Data are representative of results from at least two experiments. Statistical significance was calculated using unpaired Student’s *t* tests. *, *P < *0.05; **, *P < *0.005 (in all panels). Download FIG S2, PDF file, 0.2 MB. Copyright © 2018 Lee et al. 2018 Lee et al. This content is distributed under the terms of the Creative Commons Attribution 4.0 International license.

More importantly, B. fragilis colonization in the TLR2-deficient mice did not prevent development of AOM/DSS-induced CRC. We found that the percentage of weight loss in B. fragilis-colonized TLR2-deficient mice was comparable to that in control TLR2-deficient mice during the three periods of DSS treatment and at the endpoint of the experiment. The number of tumors and the dysplasia score for the *Tlr2^−^*^/^*^−^* mice treated with B. fragilis were also comparable to those determined for the control *Tlr2^−^*^/^*^−^* mice on day 81 of AOM-DSS treatment (Fig. 4). These findings suggest that the protective role of B. fragilis against colitis-associated CRC is partly dependent on TLR2 signaling.

## DISCUSSION

As the rate of CRC is rapidly increasing worldwide, there have been significant efforts to understand and overcome CRC pathogenesis. A number of clinical reports and laboratory models have confirmed that microbiota is a key factor in modulating chronic inflammation in the colon and CRC pathogenesis (6, 7, 29, 30). In the present study, we provided direct evidence *in vivo* that B. fragilis plays a suppressive role in the development of colitis-associated CRC. Our findings indicate that B. fragilis colonization prevents colitis-associated CRC via inhibition of CCR5 expression in the colon. Further, these findings suggest that B. fragilis is involved in a distinct preventive mechanism against intestinal inflammation in carcinogen/DSS-induced CRC compared to regulation by IL-10 or Foxp3 in T-cell transfer or a TNBS colitis model. It was recently reported that B. fragilis protects against DSS-induced colitis in germfree mice by inducing IL-10 expression (31, 32), suggesting that B. fragilis helps to prevent intestinal diseases in both the presence and the absence of microbiota, using different molecular mechanisms. Although the molecular mechanisms of this B. fragilis-mediated suppression of disease development differ depending on the experimental setting, the main common function of B. fragilis is suppression of excessive gut inflammation. Interestingly, it was suggested that CCR5 inhibition reduces infiltration of both innate and adaptive immune cells, which leads to reduced inflammation in the colon during colitis and CRC (23, 33, 34), although CCR5 might interact with other ligands, based on the comparable levels of expression of CCL3. Moreover, CCR5 blockade is under clinical trial for CRC therapy (35). Our findings indicate that the human commensal B. fragilis regulates a specific chemokine receptor, providing an additional mechanism to control intestinal inflammation and colon tumorigenesis.

Associations between dysbiosis of microbiota and increased CRC incidence have been reported previously (6, 7, 16). Disruption of microbiota in the gastrointestinal tract often leads to excessive inflammation; thus, chronic inflammation can increase the risk of CRC development (2, 4). Most beneficial bacteria in animal disease settings exert their effects via suppression of inflammatory reactions (10). In the carcinogen/DSS-induced model, B. fragilis colonization under conditions of continuous administration during CRC development seems to exert a repressive effect on uncontrolled inflammation. While the inhibition of pathological consequences is clearly dependent on the bacterial molecule PSA produced by B. fragilis ΔPSA, the administered bacteria may also indirectly drive repositioning of the microbiota in a less inflammatory manner, consequently leading to less-pathological CRC. Defining the interactions and influences among microbiota will require further investigations.

It has been of interest to investigate the contribution of TLR signaling in colon pathogenesis, as the microbiota either increases or reduces intestinal inflammation (6, 7). Admittedly, the role of the TLR pathway in tumorigenesis is complex and several contradictory results in myeloid differentiation factor 88 (MyD88)-deficient mice have been reported previously (36–38). In recent studies using AOM/DSS models, protective effects of TLR2 against colon tumorigenesis were reported (25). It was also determined that the suppressive function of B. fragilis is mediated by TLR2 signaling during the development of several diseases, including colitis and nonintestinal diseases (18, 19, 27, 28, 39). However, the importance of TLR2 signaling by B. fragilis has not been proven in colitis-associated CRC. Our current study effectively shows that human commensal B. fragilis utilized TLR2 for its protective role against the development of colitis-associated colon cancer in accordance with previous reports. Further, our report provides an important contribution to understanding the molecular mechanism of microbiota function against CRC and may lead to development of new treatments for colon tumorigenesis.

## MATERIALS AND METHODS

### Mice.

C57BL/6 mice were purchased from Taconic Biosciences. *Tlr2^−^*^/^*^−^* mice were originally purchased from Jackson Laboratories and were maintained following cohabitation with C57BL/6 mice. All procedures were performed according to guidelines of the Institutional Animal Care and Use Committee at the California Institute of Technology.

### Bacterial preparation and colonization.

B. fragilis NCTC9343 or B. fragilis ΔPSA (40, 41) bacteria were grown anaerobically at 37°C in a brain heart infusion broth (BD Difco) supplemented with 5 μg/ml hemin (Sigma-Aldrich) and 0.5 μg/ml vitamin K (Sigma-Aldrich). Mice were orally administered 5 × 10^9^ to 7 × 10^9^ CFU of B. fragilis or B. fragilis ΔPSA three times a week starting a week prior to DSS (for acute DSS-induced colitis) or AOM (for AOM-DSS CRC) administration until the end of the experiment.

### Establishment of DSS-induced colitis model.

DSS with a molecular weight of between 36,000 and 50,000 (MP Biomedicals) was administered in mice (5 mice per group) via autoclaved drinking water for 6 days followed by administration of regular drinking water. Mice were monitored and weighed daily for signs of disease. On day 8, the colons of mice from all groups were extracted, fixed immediately in 10% formalin, and stained with hematoxylin and eosin to determine disease severity. Samples were coded and scored by a pathologist in a blind fashion. The histological score was determined based on severity scores of 1 to 3 for inflammation, ulceration, crypt loss, edema, and extent of injury.

### Establishment of AOM/DSS-induced model of colon cancer.

AOM (Sigma-Aldrich) was administered in mice intraperitoneally (10 mg/kg of body weight), and DSS was administered via autoclaved drinking water for 6 days on day 5 post-AOM injection (8 mice per group). The mice underwent a second DSS administration cycle on day 25 for 6 days and a third cycle on day 55 for 4 to 6 days. On day 81, the colons of mice from all groups were extracted and cut longitudinally. The number and size of tumors in the proximal and distal portions of the colons were determined, and an approximately 1-cm length of tissue was prepared for RNA extraction. The colons were fixed immediately in 10% formalin and processed for histopathological analyses. Samples were coded and scored by a pathologist in a blind fashion. Scores of hyperplasia and dysplasia were assigned as follows: not present, 0; >0% to ≤25% of mucosa affected, 1; 26% to 50% of mucosa affected, 2; 51% to 75% of mucosa affected, 3; ≥75% of mucosa affected, 4.

### Gene expression analysis.

Colon tissue samples were homogenized using BeadBeater (BioSpec), and total RNA was extracted using an RNeasy minikit (Qiagen) and RNase-free DNase set (Qiagen) following the manufacturer’s protocols. cDNA synthesis was performed using an iSCRIPT cDNA synthesis kit (Bio-Rad). Real-time PCR (RT-PCR) was performed using an ABI Prism analyzer and SYBR master mix (Life Technologies) with primer pairs specific to cDNAs of *Il1b*, *Il6*, *Tnfa*, *Il10*, *Foxp3*, *Mcp1*, *Mip2*, *Kc*, *iNos*, *Ccl3*, and *Ccr5*. The primer sequences are listed in Table 1. Expression of the target genes was determined by comparing the relative levels of expression after normalization to the β-actin level.

**TABLE 1:** List of primer sequences for RT-PCR

Gene product	Forward primer	Reverse primer
Il1b	GCAACTGTTCCTGAACTCAACT	ATCTTTTGGGGTCCGTCAACT
Il6	TAGTCCTTCCTACCCCAATTTCC	TTGGTCCTTAGCCACTCCTTC
Tnfa	ACGGCATGGATCTCAAAGAC	GTGGGTGAGGAGCACGTAGT
Il10	CCTCAGGATGCGGCTGAG	GCTCCACTGCCTTGCTCTTATT
Foxp3	TGCGACCCCCTTTCACCT	AGTGTCCTCTGCCTCTCCGG
Mcp1	TCTCACTGAAGCCAGCTCTCTCT	CAGGCCCAGAAGCATGACA
Mip2	CATTCGCTAATTCACTGTAA	GTAGCTAGTTCCCAACTCAC
Kc	CTGGGATTCACCTCAAGAAC	GAAGCCAGCGTTCACCAGAC
iNos	CCTGGTACGGGCATTGCT	GCTCATGCGGCCTCCTT
Ccl3	TTCTCTGTACCATGACACTCTGC	CGTGGAATCTTCCGGCTGTAG
Ccr5	GTTCCTGAAAGCGGCTGTAAA	GCAGTCAGGCACATCCATAGAC

### Immunohistochemistry.

Slides of colon from mice were incubated with rabbit anti-CCR5 primary antibody (Bioss Antibodies) prepared in blocking solution (10% horse serum, 0.1% Triton X-100, 0.02% sodium azide, phosphate-buffered saline [PBS]) followed by incubation with a fluorescent dye-conjugated donkey anti-rabbit secondary antibody (Molecular Probes). DAPI (4′,6-diamidino-2-phenylindole) (Life Technologies) staining was performed following the manufacturer’s protocol, and fluorescence images were obtained using a Diaphot 300 fluorescence microscope (Nikon) with SPOT software (Sterling Heights).

### Statistical analysis.

Statistical significance was calculated by unpaired Student's *t* tests or nonparametric Mann-Whitney tests as appropriate, using Prism software (GraphPad). A *P* value of ≤0.05 was considered to indicate statistical significance of data and is referred to as such in the text. Unless otherwise specified, all data presented are representative of results from at least two similar experiments.
