# Supplementary figures and images for: The Protective Role of Bacteroides fragilis in a Murine Model of Colitis-Associated Colorectal Cancer
Source: mSphere. 2018 Nov 14;3(6):e00587-18. doi: 10.1128/mSphere.00587-18 (PMC6236802; doi:10.1128/mSphere.00587-18)

**A**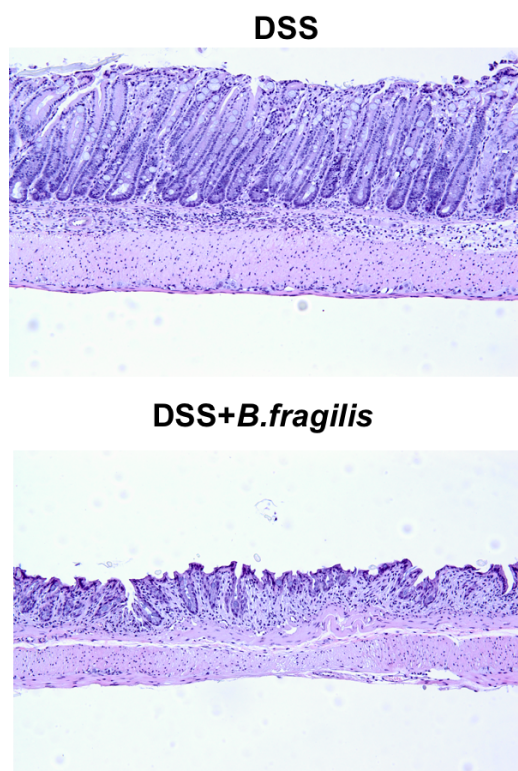**B**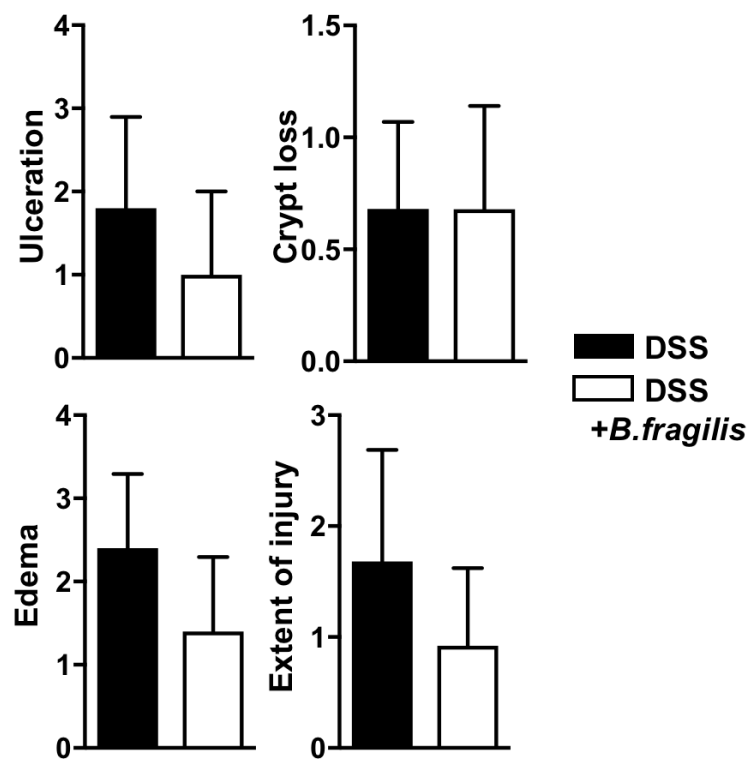**FIG S1**

Supplement: FIG S1 [file sph006182701sf1.pdf]

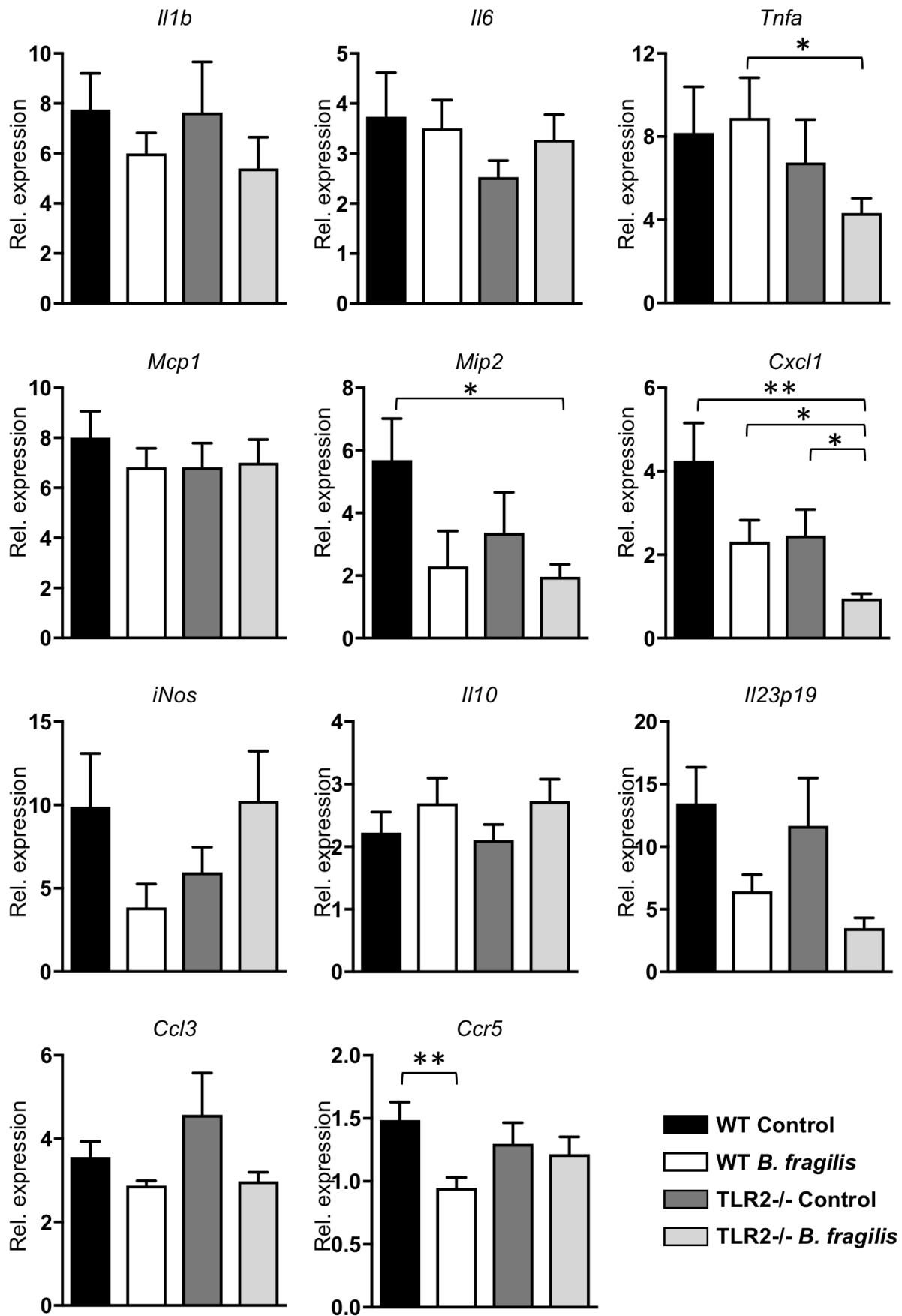

FIG S2

Supplement: FIG S2 [file sph006182701sf2.pdf]
